# Myocardial flow reserve estimation from standardized uptake values of ^13^N-ammonia imaging with a silicon photomultiplier positron emission tomography/computed tomography system

**DOI:** 10.1007/s12149-026-02219-8

**Published:** 2026-05-08

**Authors:** Masateru Kawakubo, Yoko Kaimoto, Atsushi Yamamoto, Masaki Watanabe, Akiko Sakai, Michinobu Nagao

**Affiliations:** 1 Faculty of Science and Engineering Department of Mathematics, Information Science and Engineering, Saga University, Saga, Japan; 2 Department of Health Sciences, Faculty of Medical Sciences, Kyushu University, Fukuoka, Japan; 3 Department of Radiological Services, Tokyo Women’s Medical University Hospital, Tokyo, Japan; 4 Department of Diagnostic Imaging and Nuclear medicine, Tokyo Woman’s Medical University, 8-1 Kawada-cho, Shinjuku, Tokyo, 162-8666 Japan; 5 Department of Cardiology, Tokyo Woman’s Medical University, Tokyo, Japan

**Keywords:** Myocardial flow reserve, Standardized uptake value, Positron emission tomography

## Abstract

**Objective:**

To assess the feasibility of estimating myocardial flow reserve (MFR) from static standardized uptake values (SUVs) derived using stress and rest ^13^N-ammonia positron emission tomography (PET) images acquired with a silicon photomultiplier-based PET/computed tomography (CT) system. The goal was to simplify MFR assessment by avoiding dynamic acquisition while maintaining diagnostic accuracy.

**Methods:**

We retrospectively included 121 consecutive patients who underwent ^13^N-ammonia PET myocardial perfusion imaging. The mean SUVs of the left and right ventricles were measured on transverse-axis slices corresponding to the largest cross-sectional area, and the stress-to-rest SUV ratio was calculated. A logarithmic transformation was applied to the MFR, and linear regression models between the log-transformed MFR and the stress-to-rest left ventricular-SUV ratio were developed using six-fold cross-validation across 121 patients. The predicted MFR values from the test folds were then combined to evaluate model performance across all patients with a comparable MFR distribution. Model performance was assessed using the root mean square error (RMSE) between the estimated and true MFR values, and the diagnostic performance for identifying MFRs < 2.0 by receiver operating characteristic (ROC) curve analysis with 95% confidence intervals.

**Results:**

The RMSE of the predicted MFR calculated using the linear regression model was 0.39. Using an optimal cut-off value of 1.79 for the predicted MFR, the model achieved an area under the ROC curve of 0.89 (95% confidence interval: 0.83–0.95), with a sensitivity of 75% and a specificity of 88%. The proposed simplified SUV-based approach exhibited clinically acceptable error for MFR estimation and could effectively identify patients with an impaired MFR.

**Conclusion:**

This method may facilitate quantitative MFR assessment in clinical settings where dynamic PET protocols or dedicated software are not available, thereby broadening access to physiological evaluation of myocardial perfusion.

## Introduction

^13^N-ammonia positron emission tomography (PET), with its high spatial resolution and excellent myocardial uptake properties, provides superior image quality and enhanced contrast between normoperfused and ischemic myocardial tissues [1, 2]. These characteristics are further enhanced in silicon photomultiplier (SiPM)-based PET/computed tomography (CT) systems, which offer improved detector sensitivity, timing resolution, and signal-to-noise ratios [3, 4]. This high contrast facilitates the detection of subtle perfusion abnormalities, including those associated with coronary microvascular dysfunction. Quantification of myocardial blood flow (MBF) is a central advantage of cardiac PET imaging, enabling noninvasive assessment of coronary endothelial function beyond traditional visual interpretation [5, 6]. In ^13^N-ammonia PET, MBF is estimated using kinetic modeling of time-activity curves (TACs) derived from the left ventricular (LV) myocardium and blood pool shortly after tracer injection [7]. This process can be automated with high accuracy using dedicated software. Myocardial flow reserve (MFR), defined as the ratio of stress to resting MBF, is an index of coronary vasodilator capacity that allows for the detection of multivessel disease and assessment of ischemia severity. Both stress MBF and stress MFR are useful in the evaluation of coronary artery disease (CAD) and prognosticating adverse cardiovascular outcomes [8–11]. In clinical practice, MBF quantification relies on kinetic modeling using an estimated input function, typically derived from the TAC of the LV blood pool, rather than from arterial blood sampling or direct dose measurement [7]. The TAC is reconstructed from list-mode PET data using short-frame durations during the early phase after tracer injection. However, the accuracy of the arterial input function may be affected by the injected dose and the rapid transit of high radioactivity through the LV during the initial frames. Although dynamic acquisition protocols enable accurate estimation of the arterial input function, they are often time-consuming and less feasible in routine clinical settings. Therefore, simplified approaches that maintain diagnostic performance while reducing procedural complexity are warranted.

In addition to dynamic quantification techniques, the standardized uptake value (SUV) has long served as a widely used semi-quantitative index in PET imaging across various organ systems [12, 13]. Its simplicity, reproducibility, and applicability to static images make it a practical metric in routine clinical practice. In the context of cardiac PET, myocardial SUV may also reflect the underlying perfusion and tracer extraction dynamics under certain physiological and technical conditions. Given the potential use of the SUV as a semi-quantitative surrogate, we hypothesized that the MFR could be estimated from SUV metrics obtained from static ^13^N-ammonia PET images. If feasible, this approach would allow for a simplified protocol without the need for dynamic acquisition or complex kinetic modeling, thus reducing both acquisition time and post-processing burden. Therefore, this study aimed to investigate the feasibility of estimating the MFR from static SUV values derived from stress and other ^13^N-ammonia PET images acquired using a SiPM-based PET/CT system.

## Materials and methods

### Study population

This multi-center retrospective observational study was comprehensively approved by the Institutional Review Board of Kyushu University (25101) and was conducted in compliance with the 1964 Declaration of Helsinki and all subsequent revisions. The requirement for written informed consent was waived. A total of 121 consecutive patients (94 men and 27 women; median age, 68 years; range, 40–88 years) who underwent myocardial blood flow imaging using ^13^N-ammonia PET with the Biograph Vision PET/CT system between January and June 2024 were retrospectively included in this study.

### PET/CT scanner

All data were obtained using a BioGraph Vision scanner (Siemens Healthineers, Erlangen, Germany), a fully 3D mode acquisition and time-of-flight (TOF) system, combined with a 64-slice CT scanner. The PET unit contains eight rings based on 38 detector blocks in each module, with a total of 60800 lutetium-based scintillators with 3.2×3.2×20 mm Lu_2_SiO_5_ crystals. The vision enables an axial field of view (FOV) of 26.3 cm and a transaxial FOV of 70 cm. The ring diameter was 780 mm. The coincidence window was 4.7 ns, and the lower- and higher-energy windows were 435 and 585 keV, respectively. The TOF timing resolution was 210 ps. Herein, a sequential CT scan (120 kV, 20 mA, 3 mm slice collimation) was employed. All emission data were acquired in the 3D list mode. Data were reconstructed with TrueX+TOF (ultra-HD PET), with iterations of 4, 5, 220, and 4 mm.

### ^**13**^**N-Ammonia PET imaging protocol**

Before imaging, the patient was positioned on the scanner bed, venous access was established, and blood pressure measurement and electrocardiography (ECG) were performed. A respiratory-gating sensor was used to enable synchronized acquisition. Given the short physical half-life of ^13^N-ammonia (approximately 10 min), all procedures were coordinated with radiotracer production in an on-site hot laboratory. Once the preparations were complete, resting acquisition was initiated simultaneously with the intravenous administration of approximately 185 MBq of ^13^N-ammonia. PET scans were performed in list mode for 10 min; the acquired data were reconstructed into dynamic frames during the first 2 minutes and into a static image for the remaining 8 minutes. Immediately after imaging, pharmacological stress was induced using a vasodilator administered at 0.12 mg/kg/min for 6 minutes. Three minutes after the initiation of the stress agent infusion, a second dose of ^13^N-ammonia (approximately 555 MBq) was administered, followed by list-mode PET acquisition under stress conditions. The entire procedure, including patient preparation, spanned approximately 40 minutes.

### Quantitative analysis of MBF

All image analyses and MBF quantification were performed using Syngo MBF software (Siemens Healthineers), as described previously [14]. The workflow included the automatic reorientation of images, extraction of mean myocardial and cavity time–activity curves, and generation of polar maps of absolute MBF and MFR. Left ventricular contours were detected automatically with minimal observer intervention. Using a 3-compartment kinetic model for ^13^N-ammonia, stress and rest myocardial blood flow values [mL/g/min] were computed for each polar map segment based on the resulting time–activity curves, enabling global quantification. Perfusion parameters were calculated both globally (entire left ventricle) and regionally (three coronary artery territories). Residual activity correction was applied to dynamic images acquired during pharmacological stress to exclude the influence of residual ^13^N-ammonia activity from the rest phase. Specifically, dynamic datasets were corrected by subtracting the residual activity present in the first frame of the stress acquisition (obtained immediately before stress) from the time–activity curves using the residual activity correction method implemented in the software. The MFR was calculated as the ratio of the stress MBF to the remaining MBF.

### SUV measurements

**Fig. 1:**
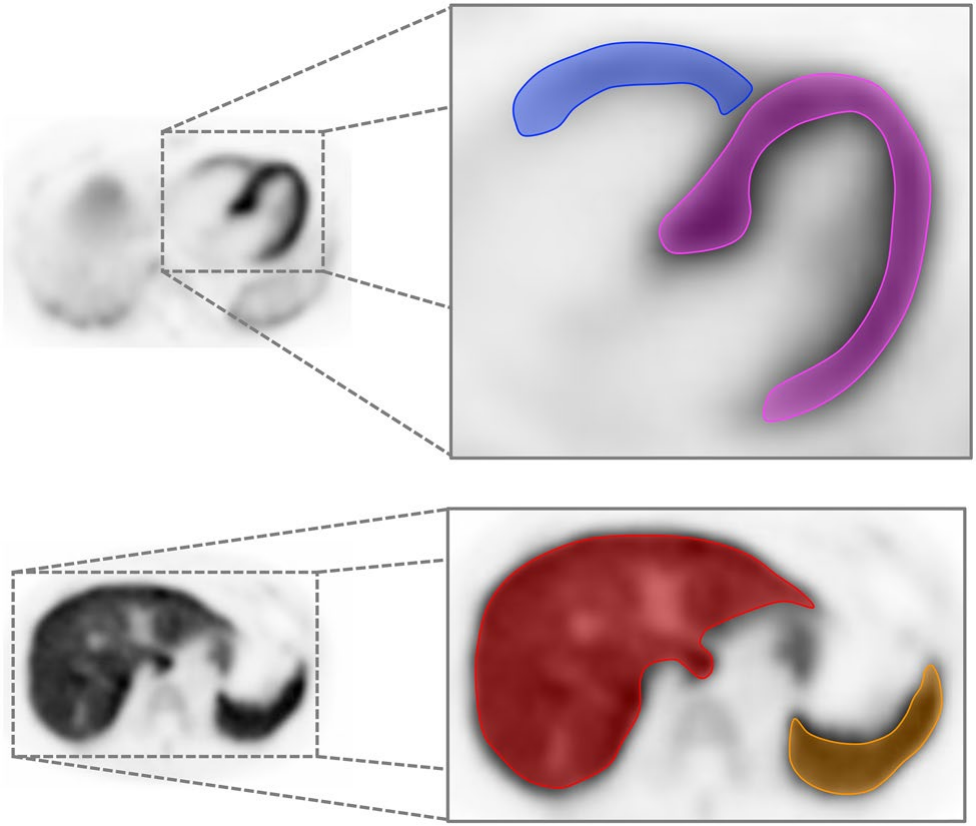
Manual delineation of regions of interest (ROIs). Representative examples of manually delineated ROIs. In the upper image, the myocardium of the left ventricle is shown in magenta, and the right ventricle is shown in blue. In the lower image, the liver and spleen are delineated in red and yellow, respectively

SUV measurements on static images were conducted using ShadeQuest/Report software (FUJIFILM Healthcare). Static PET images acquired at rest and during adenosine-induced stress were analyzed to quantify SUVs in the LV myocardium, right ventricular (RV) myocardium, liver, and spleen (Fig 1). The regions of interest (ROIs) for the LV and RV myocardium were manually delineated on the transverse-axis slices corresponding to the largest cross-sectional area. To define the myocardial contours, a relative threshold of ≥10% of the maximum uptake was applied. This threshold was determined based on the background activity measured in the lung fields, where the background noise corresponded to an SUV of approximately 2.0, allowing for good anatomical delineation of the myocardium. For the liver and spleen, ROIs were manually placed on a horizontal long-axis slice where each organ was clearly visualized. The representative SUV values in each region were defined as the mean values in each ROI. Additionally, the stress-to-rest SUV ratio was calculated for each region and served as a surrogate marker analogous to the MFR.

### Feature selection, regression, and model evaluation for predicting MFR

First, the correlation coefficients between each feature and MFR were calculated to assess their univariate associations. Subsequently, multivariate regression analysis was performed using a generalized linear model with a gamma distribution and log link function, and effect ratios (exp[β]) with 95% confidence intervals were computed to quantify the contribution of each feature to MFR. Only features showing statistical significance in the multivariate regression (*P* < 0.05) were regarded as relevant contributors to MFR.

To ensure an even representation across the spectrum of MFR values, the full dataset of 121 patients was divided into six folds based on quantiles of the MFR distribution using qcut. Six-fold cross-validation was then performed such that each fold served once as the test set while the remaining folds were used for training, ensuring that the MFR distribution was preserved across all folds. A logarithmic transformation was applied to the MFR, and six-fold cross-validated linear regression models were constructed using only statistically significant features. For each fold, the regression slope and intercept were estimated, and the corresponding regression lines were plotted over the observed data to visualize fold-wise predictive performance.

For each fold, a linear regression model was trained on the training set using statistically significant features with a logarithmic transformation of MFR. The estimated slope and intercept from the training set were then applied to the corresponding test set to predict the log-transformed MFR.

### Statistical analysis

Spearman’s correlation coefficients between the observed MFR and the predicted MFR values were calculated. The accuracy of the predicted MFR was evaluated by calculating the root mean square error (RMSE) between the predicted and reference MFR values. Furthermore, the diagnostic performance of the predicted MFR in identifying cases with impaired flow reserve (MFR<2.0) was assessed using receiver operating characteristic (ROC) curve analysis.

## Results

### Dataset characteristics

Baseline characteristics of the study population are summarized in Table 1. Patients were stratified into two groups according to MFR (<2.0 vs ≥2.0), and their clinical characteristics were compared. Patients with MFR < 2.0 were significantly older and had lower hemoglobin levels than those with MFR ≥ 2.0. In addition, resting heart rate was significantly higher in patients with MFR < 2.0. Regarding LV function, patients with MFR < 2.0 showed higher stressed end-systolic volume and lower stressed ejection fraction, indicating impaired systolic function under stress conditions. No significant differences were observed in body size parameters (height, weight, BMI). The prevalence of cardiovascular risk factors (hypertension and diabetes mellitus), smoking status, and advanced chronic kidney disease tended to be higher in patients with MFR < 2.0, whereas dyslipidemia was slightly more prevalent in patients with MFR ≥ 2.0. As expected, stress MBF, resting MBF, and global MFR were significantly different between groups.

**Table 1:** Patients characteristics

Characteristic	Total	MFR<2.0	MFR≥2.0	*P*-value
No. of patients	121	69	52	−
Male (%)	94 (78)	56 (81)	38 (73%)	−
Age [years-old]	69 (60-76)	73 (62-80)	67 (58-73)	0.006
Hight [cm]	165 ± 9	165 ± 9	165 (161-170)	0.729
Weight [kg]	66 (57-74)	64 (57-72)	67 ± 12.	0.441
BMI [kg/m^2^]	23.8 (21.6-26.1)	23.3 (21.0-25.6)	24.6 ± 3.4	0.099
Hemoglobin [g/dL]	13.4 ± 1.7	13.0 ± 1.9	13.9 ± 1.2	0.006
Hypertension (%)	73 (60)	43 (62)	30 (58)	−
Diabetes mellitus (%)	54 (45)	37 (54)	17 (33)	−
Dyslipidemia (%)	86 (71)	47 (68)	39 (75)	−
Advanced CKD (G4–G5) (%)	22 (18)	17 (25)	5 (10)	−
Smoker (%)	56 (46)	36 (52)	20 (38)	−
Current	16	9	7	−
Former	40	27	13	−
Never	65	33	32	−
Stressed HR [bpm]	78.9 ± 13.5	78.5 ± 15.4	79.5 ± 10.6	0.696
Resting HR [bpm]	67.0 (60.0-77.0)	69.0 (64.0-77.0)	66.0 (57.8-76.5)	0.022
Stressed LVEDV [mL]	78.9 ± 13.5	78.5 ± 15.4	79.5 ± 10.6	0.696
Resting LVEDV [mL]	67.0 (60.0-77.0)	69.0 (64.0-77.0)	66.0 (57.8-76.5)	0.022
Stressed LVESV [mL]	44.0 (31.0-75.0)	49.0 (32.0-81.0)	40.0 (27.8-54.8)	0.038
Resting LVESV [mL]	99.0 (81.0-127.0)	102.0 (82.0-136.0)	95.0 (75.2-116.2)	0.132
Stressed LVEF [%]	38.0 (26.0-64.0)	44.0 (29.0-70.0)	33.0 (21.0-48.2)	0.028
Resting LVEF [%]	110.0 (90.0-143.0)	116.0 (92.0-150.0)	107.0 (86.8-132.2)	0.282
Stressed MBF [mL/g/min]	1.7 ± 0.6	1.4 (1.0-1.8)	2.1 ± 0.5	<0.001
Resting MBF [mL/g/min]	0.9 (0.8-1.1)	0.9 (0.8-1.1)	0.9 ± 0.2	0.025
Global MFR	1.9 (1.4-2.3)	1.4 (1.2-1.7)	2.5 (2.2-2.7)	<0.001

Variables that followed a normal distribution, as determined by the Shapiro–Wilk test, are presented as mean ± standard deviation, while non-normally distributed variables are expressed as median (interquartile range, 25th–75th percentile). P-values indicate the comparison between patients with MFR < 2.0 and those with MFR ≥ 2.0.

BMI: body mass index, CKD: chronic kidney disease, HR: heart rate, LVEDV: left ventricular end-diastolic volume, LVESV: left ventricular end-systolic volume, LVEF: left ventricular ejection fraction, MBF: myocardial blood flow, MFR: myocardial flow reserve.

### Selection of SUV-derived features and MFR regression

**Fig. 2:**
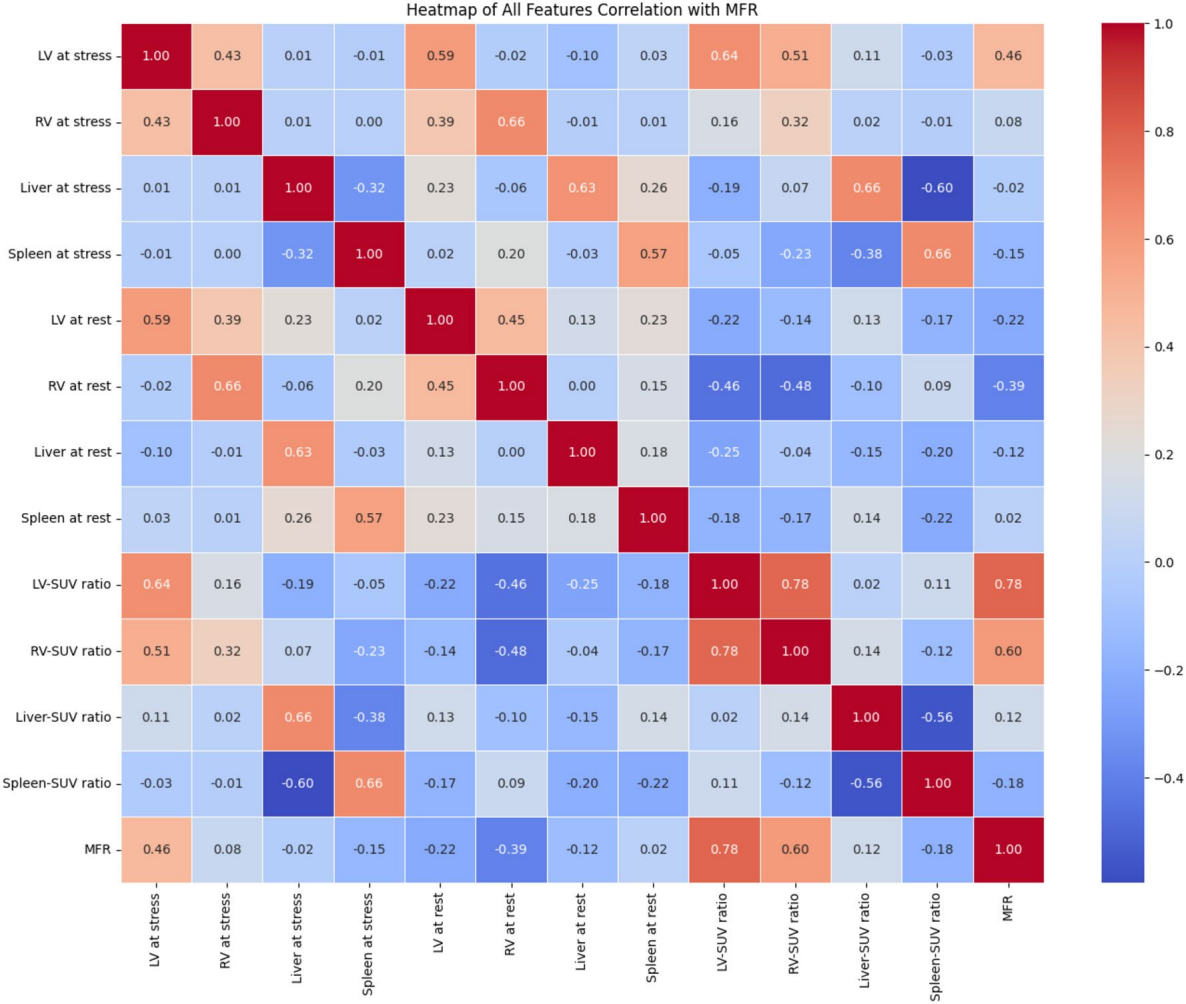
Heatmap of correlations among all features. Pearson’s correlation coefficients are shown. LV: left ventricle, RV: right ventricle, SUV: standardized uptake value, MFR: myocardial flow reserve

Figure 2 shows a heat map of the Pearson’s correlation coefficients (*r*) among all SUV-derived features. The LV-SUV ratio demonstrated a strong correlation (*r* = 0.78) with respect to MFR. The RV-SUV ratio and LV-SUV at the stress state showed moderate correlations with MFR (*r* = 0.60 and *r* = 0.46, respectively). The RV-SUV at the resting state was negatively correlated with MFR (*r* = −0.39). Other SUV-derived features showed weak associations with MFR, with correlation coefficients below 0.2 in either direction, indicating low relevance to MFR. Table 2 shows the results of the multivariate regression analysis for all 121 patients. Among all evaluated features, only the LV-SUV ratio was significantly associated with MFR, with an effect ratio of 2.65 *(P* = 0.007), whereas no other features reached statistical significance. Based on these results, the LV-SUV ratio was selected as the sole feature for constructing the linear regression model to predict MFR.

**Table 2:** Multivariate regression coefficients for predicting MFR

Feature	Effect ratio (exp[β])	95% CI (Lower)	95% CI (Upper)	*P-*value
LV at stress	1.006	0.933	1.084	0.883
RV at stress	1.039	0.915	1.180	0.556
Liver at stress	0.967	0.875	1.068	0.507
Spleen at stress	0.951	0.875	1.034	0.238
LV at rest	0.969	0.862	1.090	0.598
RV at rest	0.954	0.786	1.157	0.630
Liver at rest	1.047	0.925	1.186	0.464
Spleen at rest	1.059	0.986	1.137	0.113
LV-SUV ratio	2.653	1.311	5.367	0.007
RV-SUV ratio	0.745	0.433	1.280	0.286
Liver-SUV ratio	1.160	0.618	2.178	0.644
Spleen-SUV ratio	0.899	0.407	1.989	0.793

MFR: myocardial flow reserve, CI: confidence interval, P value: probability

Regression coefficients were estimated using a gamma regression model with a log link. Exponentiated coefficients represent effect ratios, indicating the multiplicative change in MFR per one-unit increase in each explanatory variable. Effect ratios greater than 1 indicate an increase in MFR, whereas values less than 1 indicate a decrease. The intercept reflects the expected baseline MFR when all explanatory variables are zero.

**Fig. 3:**
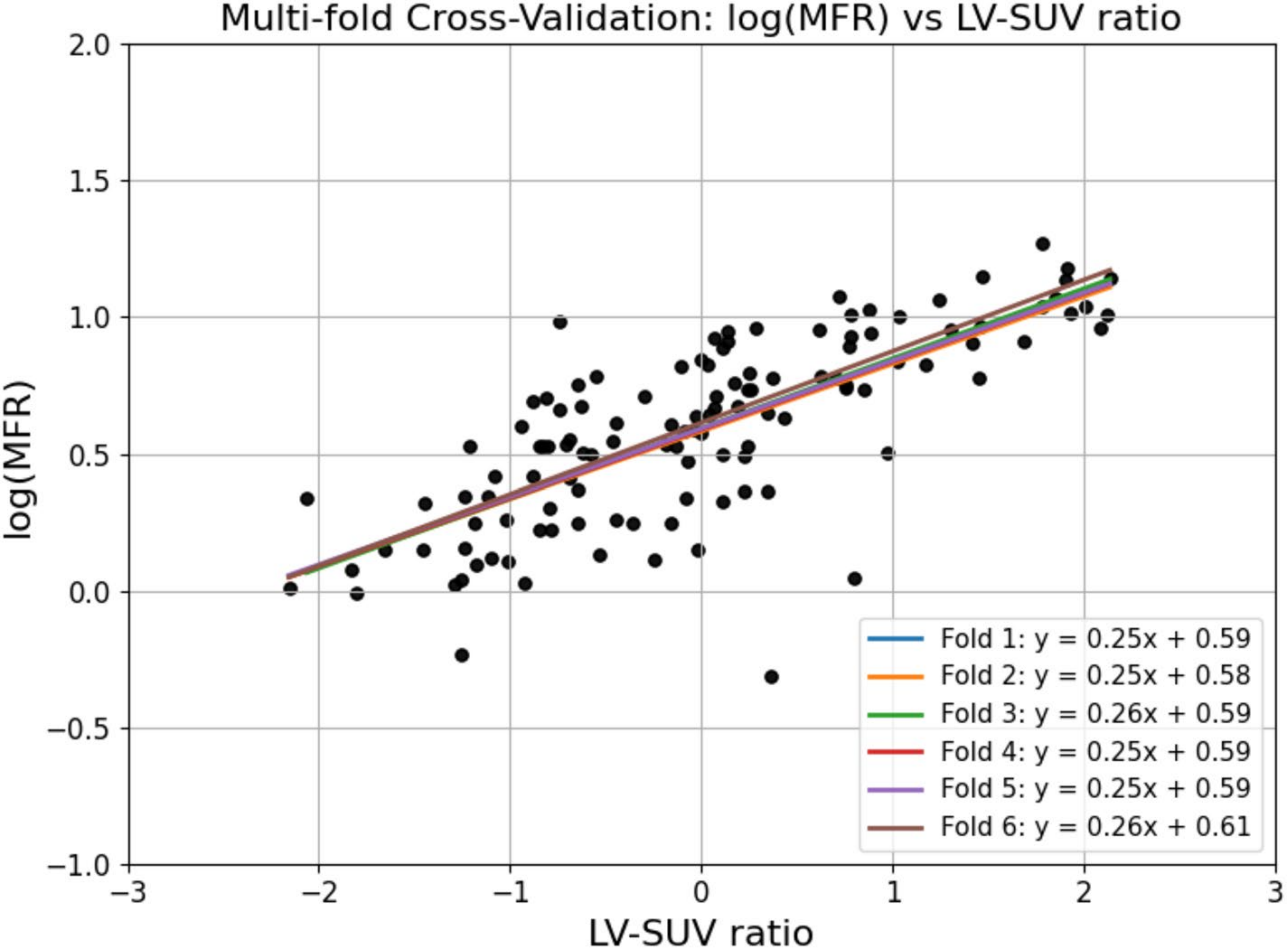
Scatter plots of the LV-SUV ratio versus the logarithmically transformed MFR for each fold in the six-fold cross-validation. For reference, the LV-SUV ratio values shown are standardized using z-score normalization (mean = 0, SD = 1). Values above 0 represent measurements higher than the mean, whereas values below 0 represent measurements lower than the mean. LV: left ventricle, RV: right ventricle, SUV: standardized uptake value, MFR: myocardial flow reserve

Figure 3 shows scatter plots of the LV-SUV ratio versus the logarithmically transformed MFR for each fold in the six-fold cross-validation, along with the corresponding regression lines. The estimated slopes of the regression lines ranged from 0.25 to 0.26, and the intercepts ranged from 0.59 to 0.61, indicating consistent regression parameters across all folds. For reference, the LV-SUV ratio values shown in Fig 3 are standardized using z-score normalization (mean = 0, SD = 1). Thus, values above 0 represent measurements higher than the mean, whereas values below 0 represent measurements lower than the mean.

### Evaluation of linear regression models for predicting MFR

**Fig. 4:**
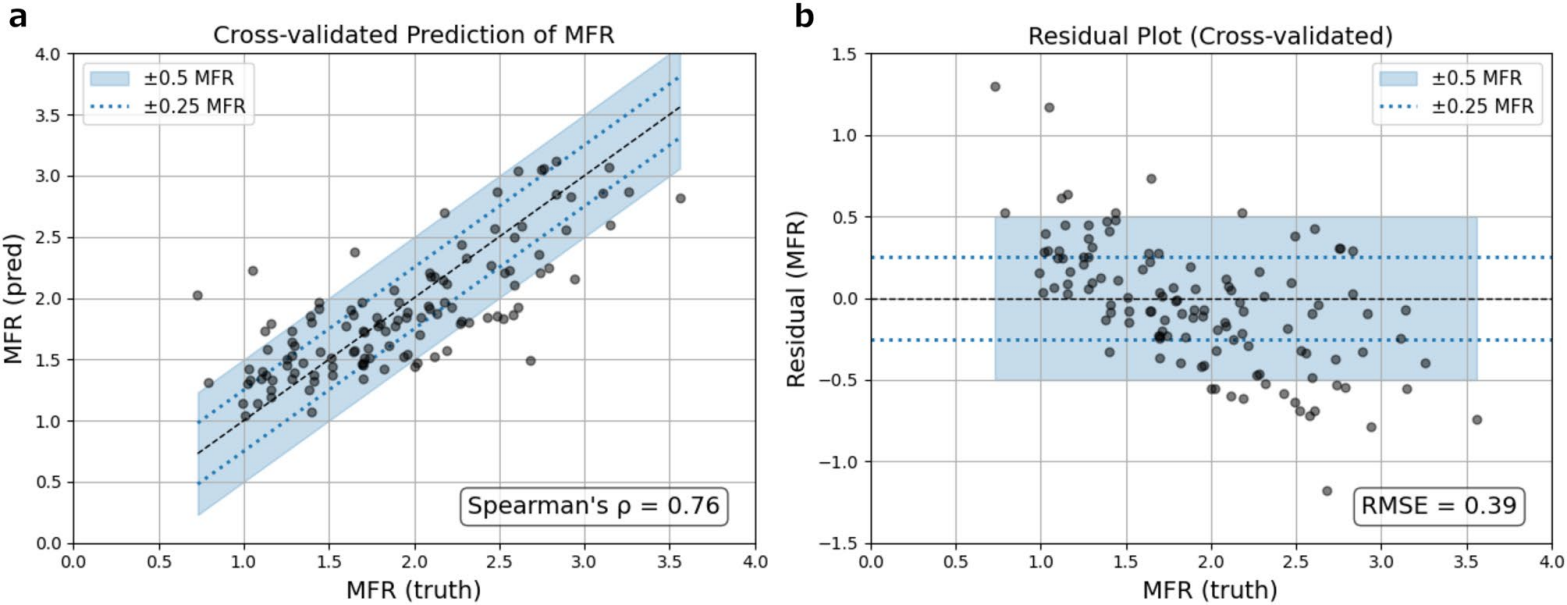
Correlation and residuals between true and predicted MFR. (**a**) Scatter plot of predicted MFR (y-axis) versus true MFR (x-axis). The blue shaded band represents the range of ±0.5 around the identity line, and the blue dotted lines indicate ±0.25. (**b**) Residual plot of predicted MFR against true MFR. The x-axis indicates the true MFR, and the residuals (true − predicted) are shown on the y-axis. The blue shaded band represents residuals within ±0.5, and the blue dotted lines indicate ±0.25. Abbreviations: MFR, myocardial flow reserve

Figure 4a shows the scatter plot of observed versus predicted MFR values across all folds, together with Spearman’s correlation coefficient (ρ = 0.76, *P* < 0.01), indicating a strong monotonic association. Figure 4b presents the residual plot, showing the difference between the predicted and observed MFR values, with a RMSE of 0.39. Among all 121 patients, 97 (80.2%) had an absolute MFR prediction error within ±0.5 (blue shaded area in the plot), and 61 (50.4%) were within ±0.25 (blue dotted lines).

**Fig. 5:**
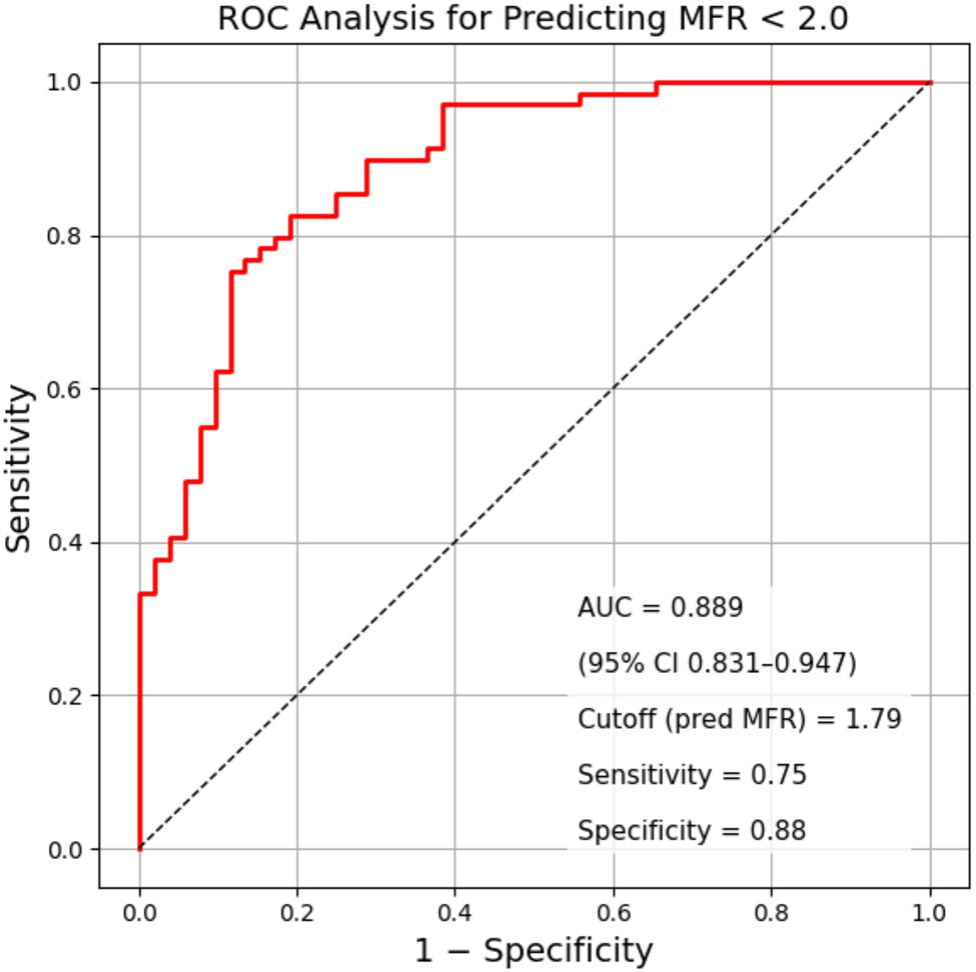
Receiver operating characteristic (ROC) curve for the diagnosis of impaired myocardial flow reserve (MFR < 2.0). The ROC curve illustrates the diagnostic performance of the proposed model in identifying cases with impaired MFR. The curve (AUC) quantifies the overall discriminative ability of the model

Figure 5 shows that, using an optimal cut-off value of 1.79 for the predicted MFR, the model achieved an area under the ROC curve of 0.89 (95% CI: 0.83–0.95), with a sensitivity of 75% and a specificity of 88%.

### Case presentation

**Fig. 6:**
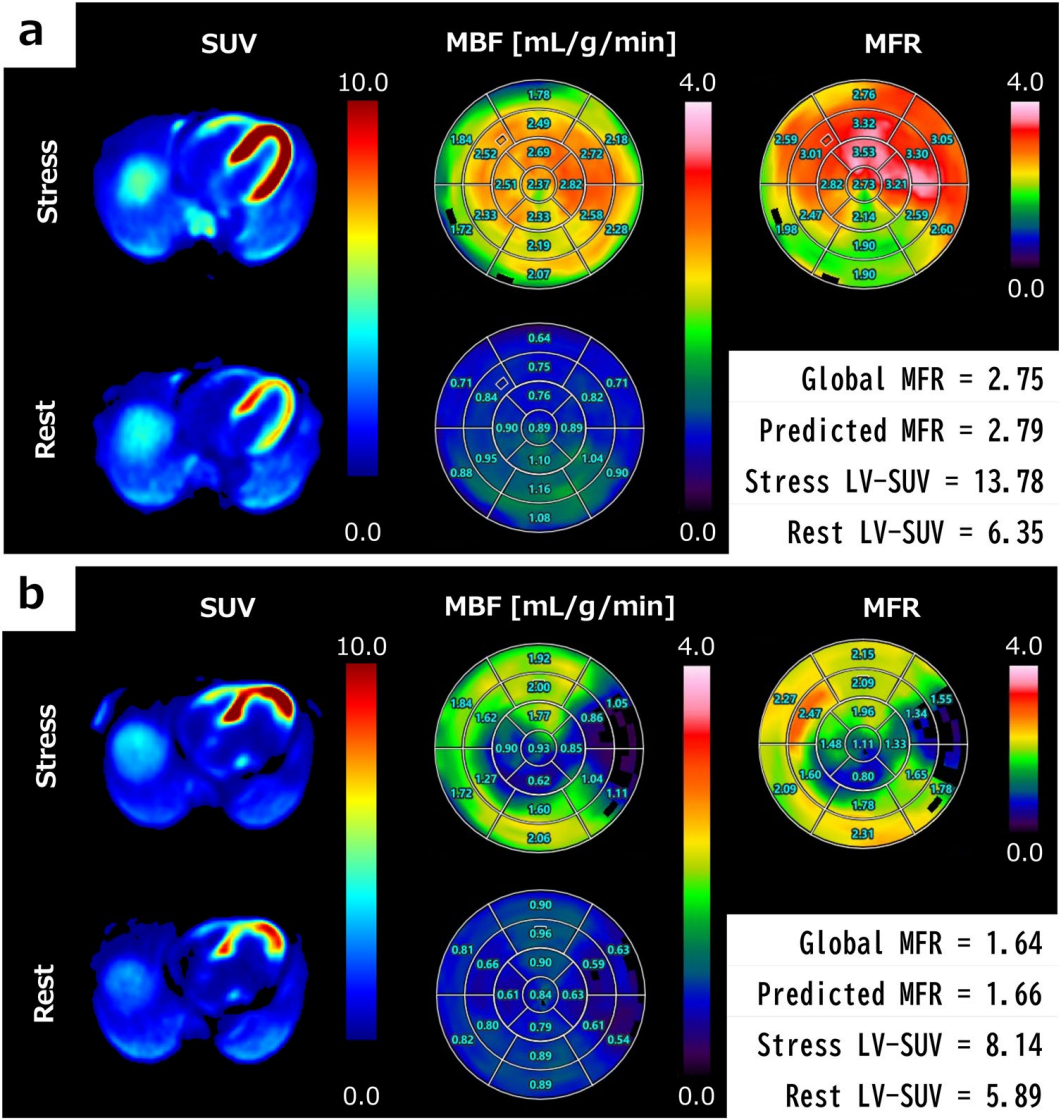
Representative cases. (**a**) A case with preserved myocardial flow reserve (MFR) (> 2.0) showing accurate prediction. (**b**) A case with reduced MFR (< 2.0) showing accurate prediction

Figure 6 shows representative cases wherein MFR was accurately predicted using the LV-SUV ratio. The case presented in Fig. 6a is that of a 58-year-old male with no coronary artery disease risk factors or anemia, who had quit smoking more than 10 years prior. The transverse SUV images demonstrated clear delineation of the left ventricle. The LV-SUV in the stress condition increased to more than twice that at rest, resulting in an accurately predicted global MFR of 2.79, which was identical to the reference value. The case presented in Fig. 6b is that of a 61-year-old male with multiple cardiovascular risk factors, including hypertension, diabetes mellitus, dyslipidemia, advanced chronic kidney disease (stage ≥ 4), and anemia. The myocardial SUV in the left ventricular free wall was markedly reduced to near-background levels in both rest and stress conditions. The LV-SUV ratio was approximately 1.4, and the reduced global MFR of 1.64 was accurately predicted as 1.66.

## Discussion

The cohort was divided into two groups using an MFR cut-off of 2.0, resulting in approximately equal group sizes. Although the dataset consisted of consecutive cases, it was considered to contain sufficient information for MFR regression. Among clinical and PET-derived parameters, patients with MFR < 2.0 were significantly older and had lower hemoglobin levels than those with MFR ≥ 2.0. In addition, resting heart rate was higher in patients with MFR < 2.0. Regarding LV function, patients with MFR < 2.0 showed higher stressed end-systolic volume and lower stressed ejection fraction, indicating impaired systolic function under stress conditions. Despite these differences, the regression slopes and intercepts across the six folds were highly consistent, indicating that the fold allocation preserved the overall MFR distribution without substantial bias, thereby allowing reliable model development and cross-validation.

Despite the well-known kinetic limitations of ^13^N-ammonia, SUV-based estimation may retain practical validity under certain physiological conditions. Although the relationship between myocardial blood flow and tracer uptake becomes non-linear and approaches saturation at higher flow levels, particularly under pharmacological stress, a monotonic relationship is generally preserved across a wide flow range. This implies that relative indices derived from SUV, such as the LV-SUV ratio, can reflect underlying flow differences, especially within low-to-moderate flow conditions where the extraction fraction remains relatively stable. However, at higher flow ranges, reduced extraction efficiency and increased washout may lead to the systematic underestimation of myocardial uptake, which could propagate into bias in MBF estimation. Importantly, when calculating myocardial flow reserve as a ratio of stress to rest values, some of these systematic effects may be partially mitigated, although not they may not be completely eliminated. Therefore, while SUV-based approaches cannot fully replace kinetic modeling, they may provide a reasonable and practical surrogate for MFR estimation within a clinically relevant range, with an understanding of their inherent physiological limitations. However, it should be recognized that MFR, as a ratio of stress to rest flow, does not fully capture the heterogeneity of coronary microvascular dysfunction. Recent studies have suggested the presence of distinct coronary microvascular dysfunction endotypes, including a “classical” phenotype characterized by reduced hyperemic flow and an “elevated resting flow” phenotype with relatively preserved stress flow [15]. Because MFR alone cannot distinguish whether an abnormal value is driven by impaired hyperemic flow or increased resting flow, SUV-derived MFR is similarly limited in its ability to differentiate these pathophysiological patterns. Therefore, while the proposed SUV-based approach may be a simplified surrogate for global flow reserve, it does not replace the need for direct quantification of both rest and stress MBF for the comprehensive characterization of coronary microvascular dysfunction. In particular, endotype-based assessment and interpretation require the separate evaluation of absolute flow values, which should be considered when applying the present method in clinical or research settings.

Among the variables analyzed, the LV-SUV ratio emerged as a strong explanatory factor for predicting the MFR. SUV, normalized to body weight, is a semiquantitative metric of tracer uptake and is widely recognized in nuclear cardiology as a reliable indicator. The LV-SUV ratio used in this study was conceptually similar to the simplified approach for estimating MFR proposed by Sherif et al., who used 18F-flurpiridaz in an animal model [16]. Although the tracers differed, both ^18^F-flurpiridaz and ^13^N-ammonia demonstrated a high degree of linearity between the administered dose and myocardial uptake. Thus, the SUV has been shown to serve as a reliable surrogate marker of MBF. Sherif et al. reported a strong correlation (r = 0.88) between SUV ratios and MBF measured using the microsphere method. These findings support the utility of the LV-SUV ratio as a robust predictor of MFR. Furthermore, by eliminating the need for dynamic scanning, these simplified protocols can enhance the accessibility of myocardial perfusion imaging, particularly considering the increasing use of fluorodeoxyglucose in clinical practice. The applicability of this approach may extend to other PET tracers such as ^82^Rb. Although ^82^Rb is characterized by a lower first-pass extraction fraction and a non-linear relationship between myocardial blood flow and tracer uptake, particularly at higher flow ranges, a monotonic relationship is generally preserved. This indicates that relative uptake indices, such as SUV ratios, may provide meaningful surrogate information for MFR estimation, albeit with potential limitations in high-flow conditions. In addition, the extremely short physical half-life of ^82^Rb (~75 s) minimizes the influence of residual activity between rest and stress acquisitions, supporting its suitability for same-day imaging protocols. Furthermore, in myocardial perfusion SPECT using ^99m^Tc-labeled myocardial perfusion tracers, the linearity between blood flow and myocardial uptake is further limited owing to lower extraction fractions and pronounced saturation effects. Nevertheless, previous studies have demonstrated that relative indices, such as the increasing ratio between rest and stress uptake, can correlate with PET-derived MFR [17]. These findings indicate that strict linearity between myocardial blood flow and tracer uptake is not a prerequisite for surrogate estimation and that a preserved monotonic relationship may be sufficient for establishing clinically useful correlations. Taken together, from perspectives of tracer kinetics and effective myocardial half-life, the proposed method may have broad applicability across different tracers and imaging modalities. Although tracer-specific characteristics should be carefully considered, the present findings support the potential for wider clinical implementation beyond ^13^N-ammonia PET.

Although multiple SUV-derived indices were examined, the LV-SUV ratio alone achieved a high level of predictive accuracy for the MFR in the linear regression analysis. Moreover, the proposed model demonstrated clinical feasibility by effectively identifying cases with MFR<2.0, which is a widely accepted threshold for impaired coronary vasodilatory capacity [18–20]. Notably, the model showed a relatively high specificity, indicating that predicted MFR values ≥2.0 reliably identified patients without impaired coronary vasodilatory function, while cases with predicted MFR <2.0 could be prioritized for further dynamic PET assessment or invasive evaluation. These findings suggest that the model can provide interpretable results without relying on complex multivariate algorithms or the inherent “black-box” nature of deep-learning models [21, 22].

The proposed regression model was able to predict MFR within an absolute error of 0.5 in approximately 80% of the cohort, while the proportion of predictions within an absolute error of 0.25 was limited to approximately 50%. This study was conducted in a single-center cohort, and potential variations across vendors or imaging protocols could not be assessed. Nevertheless, the study highlights the value of using clinically familiar features such as organ SUV values. Furthermore, multivariate analysis demonstrated that MFR could be accurately estimated using a simple linear regression based solely on the LV-SUV ratio. This approach offers greater interpretability compared with estimations relying on a large number of radiomics features that may be protocol dependent. Therefore, our straightforward linear approach is highly compatible with routine diagnostic workflows, and future studies using larger multicenter datasets may allow for the optimization of the SUV ratio–MFR relationship, potentially improving predictive performance.

This study has some limitations. SUV measurements were obtained from manually delineated ROIs on a single transverse slice corresponding to the largest cross-sectional myocardial area, rather than from the entire left ventricular myocardium. In contrast, the MFR derived from dynamic PET reflects global myocardial perfusion. Therefore, this approach may not fully capture spatial heterogeneity, and focal perfusion defects located outside the selected slice may not be reflected in the SUV measurements. This aspect represents an inherent limitation of the single-slice methodology. Furthermore, the estimated MFR demonstrated high agreement with the reference standard despite the use of a single-slice approach. These findings suggest that, in this specific population, a representative mid-ventricular slice provides a reasonable approximation of global myocardial perfusion. However, this assumption may not hold in patients with marked regional ischemia or highly heterogeneous perfusion patterns; caution is warranted when generalizing our results to such populations. Additionally, the use of manual ROI delineation may introduce operator-dependent variability. In future implementations, the left ventricular myocardial mask derived from dynamic PET during MFR calculation could be directly applied to static SUV images, enabling the automated and comprehensive quantification of whole-myocardial SUV. Such integration would eliminate the need for manual ROI placement and allow full spatial coverage of the left ventricle, further enhancing the robustness, generalizability, and clinical utility of our approach.

This study demonstrated the feasibility of estimating the MFR using static SUVs derived from ^13^N-ammonia PET images acquired with a SiPM-based PET/CT system. Among the various SUV-based indices, the LV-SUV ratio showed the strongest correlation with the MFR and enabled accurate linear prediction without the need for dynamic scanning. The proposed approach achieved clinically acceptable estimation errors and effectively identified cases with an impaired MFR (<2.0), highlighting its potential utility as a simplified and interpretable alternative to conventional dynamic PET protocols. These findings suggest that static SUV measurements may support broader clinical applications of MFR assessments, particularly in settings where dynamic imaging is not feasible. Future studies with multicenter data may further validate and optimize the use of static SUV measurements for MFR estimation.

## Data Availability

The data supporting the findings of this study are available from the corresponding author (MN) upon reasonable request. The data are not publicly available due to privacy and institutional restrictions.
